# Immobilization-Induced Hypercalcemia in COVID-19 With a Prolonged Intensive Care Unit Stay

**DOI:** 10.7759/cureus.24081

**Published:** 2022-04-12

**Authors:** Lakshmi Kannan, Rishi Raj, William Rhoad, Ramya Akella, Aasems Jacob

**Affiliations:** 1 Nephrology, Pikeville Medical Center, Pikeville, USA; 2 Endocrinology, Pikeville Medical Center, Pikeville, USA; 3 Internal Medicine, University of Pikeville Kentucky College of Osteopathic Medicine, Pikeville, USA; 4 Internal Medicine, Pikeville Medical Center, Pikeville, USA; 5 Hematology and Oncology, Pikeville Medical Center, Pikeville, USA

**Keywords:** renal replacement therapy, immobilization, physical therapy, bisphosphonates, covid-19, hypercalcemia

## Abstract

Immobilization is an uncommon etiology of hypercalcemia. It is usually seen in conditions associated with limited movements such as spinal cord injuries, vascular events, or following prolonged hospitalization. Hereby, we present a case of a young patient who had prolonged hospitalization following infection with severe acute respiratory syndrome coronavirus 2 (SARS-CoV-2). During her prolonged and complicated hospital stay, she developed severe hypercalcemia secondary to immobilization, which was resistant to treatment with hydration, calcitonin, and denosumab. One dose of zoledronic acid was used, although the patient was on hemodialysis, with adequate response in calcium levels. This case illustrates that patients with COVID-19-related hospitalization are at increased risk of immobilization-induced hypercalcemia, likely due to prolonged hospital stay due to critical illness and lack of early physical therapy during hospitalization.

## Introduction

Calcium disorders, including hypocalcemia and hypercalcemia, have been reported with coronavirus disease 2019 (COVID-19) infections [1,2] and have been associated with poor prognosis. Calcium is essential for virus structure formation and gene expression [3,4]. Immobilization-induced hypercalcemia in COVID-19 patients with prolonged intensive care unit (ICU) stay has been reported in the literature [5]. Diagnosis requires an extensive evaluation to rule out other causes of hypercalcemia [6]. Studies have shown that hypercalcemia has been associated with poor prognosis in these patients, particularly in elderly frail patients [2]. Early rehabilitation is recommended for COVID-19 patients, especially after an ICU stay, to improve functional outcomes at hospital discharge. We present a case of resistant hypercalcemia secondary to immobilization following COVID-19 infection, which is a unique complication following infection with SARS-CoV-2.

## Case presentation

A 28-year-old white female with a medical history of craniopharyngioma status post multiple brain surgeries, ventriculoperitoneal shunt secondary seizure disorder, central hypothyroidism, morbid obesity with a body mass index of 46 kg/m^2^, and type II diabetes mellitus initially presented with shortness of breath. She was found to be in acute hypoxic respiratory failure secondary to severe COVID-19. She had a complicated and prolonged hospital stay for more than 60 days and required broad-spectrum antibiotics, vasopressors, mechanical ventilation, and extracorporeal membrane oxygenation (ECMO) for septic shock. She also developed acute kidney injury on presentation requiring renal replacement therapy with intermittent hemodialysis. Eventually, she was weaned off dialysis and was stable until hospital day 32, when she developed hypercalcemia. Laboratory investigations showed elevated total calcium of 12.8 mg/dL (corrected for albumin) with suppressed parathyroid hormone (PTH) level of <6.30 pg/mL, suggestive of PTH-independent hypercalcemia. The rest of the laboratory workup is summarized in Table 1.

**Table 1:** Laboratory investigations for evaluation of hypercalcemia

Laboratory test	Reference range	Results (hospital day 32)
Total calcium	8.5-10.1 mg/dL	12.8 mg/dL
Ionized calcium	1.19-1.29 mMol/L	1.34 mMol/L
Albumin	3.5-5.5 g/dL	1.5 g/dL
Alkaline phosphatase	44-121 IU/L	229 IU/L
Estimated glomerular filtration rate	>60 mL/minute/1.73 m^2^	27.9 mL/minute/1.73 m^2^
Blood urea nitrogen	7-18 mg/dL	82 mg/dL
Creatinine	0.60-1.30 mg/dL	2.10 mg/dL
Magnesium	1.7-2.4 mg/dL	2 mg/dL
Phosphorus	2.5-4.9 mg/dL	5 mg/dL
25-Hydroxy vitamin D	30-100 ng/mL	19 ng/mL
Parathyroid hormone	11-51 pg/mL	<6.3 pg/mL
Parathyroid hormone-related peptide	<2.5 pmol/L	<2 pmol/L

**Figure 1:**
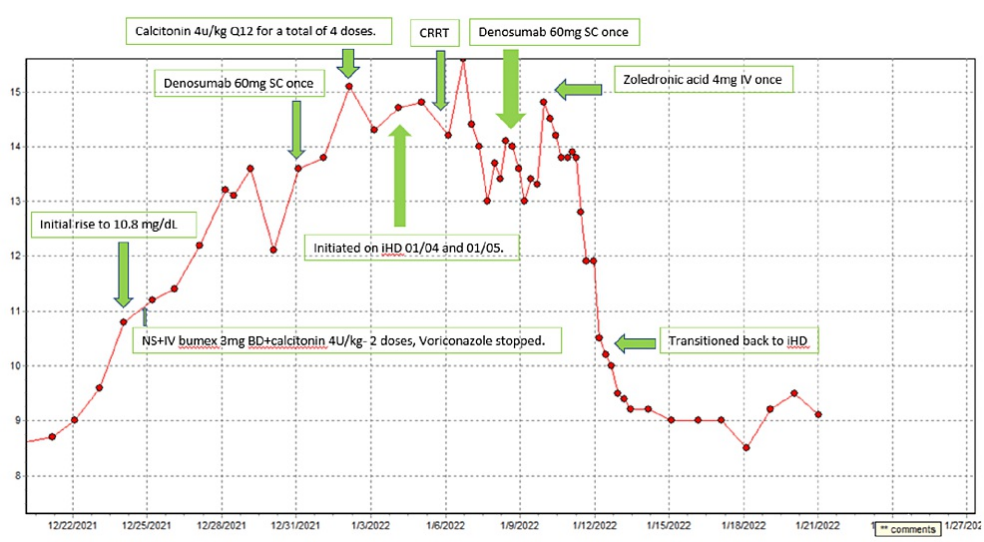
Calcium trend and treatment CRRT: continuous renal replacement therapy, SC: subcutaneous, iHD: intermittent hemodialysis, Q12: every 12 hours, BD: twice daily, NS: normal saline, IV: intravenous

Our differential diagnosis included drug-induced, malignancy, or hypercalcemia of immobilization. Extensive workup for hypercalcemia of malignancy was negative and demonstrated normal chest, abdomen, and pelvis CT, normal urine and serum electrophoresis, and negative parathyroid hormone-related peptide (PTHrP). There was no clinical or biochemical evidence to suggest any endocrinopathies such as hyperthyroidism or adrenal insufficiency. In the context of prolonged immobilization and with the absence of other underlying etiologies, hypercalcemia was attributed to immobilization. The patient was started on intravenous hydration and subcutaneous calcitonin. Moreover, due to the possible association of voriconazole with hypercalcemia, it was changed to posaconazole. Despite initial measures for 48 hours, calcium levels continued to trend up (total calcium: 14.1 mg/dL), and one dose of subcutaneous denosumab 60 mg (dose adjusted for renal function) was administered. However, her calcium continued to trend up; hence, she was restarted on hemodialysis. Calcium levels only minimally improved and stayed >14 mg/dL following two hemodialysis sessions; hence, the second dose of denosumab 60 mg was administered. She was then restarted on continuous renal replacement therapy as she became hypotensive at this point. Despite all these measures, calcium levels did not improve; hence, a single dose of intravenous zoledronic acid 4 mg was given, with a significant improvement in calcium level to 9.4 mg/dL within 72 hours. She was also started on rehabilitation with passive physical therapy. The patient is awaiting long-term acute care (LTAC) hospital placement and continues to be on intermittent hemodialysis. Her calcium continues to remain consistently between 9 mg/dL and 9.8 mg/dL. The total calcium level trend and the treatment given are summarized in Figure 1.

## Discussion

The COVID-19 pandemic has been associated with hundreds of thousands of deaths, especially in patients with comorbidities. Calcium plays an important role in viral invasion [3,4]. By infecting cells, the SARS-CoV-2 gene encodes a transmembrane protein called viroporins. Viroporins have ion nonselective channel activity for calcium, causing dysregulation of calcium homeostasis [3,4,7].

Hypercalcemia due to immobilization in hospitalized patients is often mild and resolves spontaneously [6]. However, in the case of acute and severe immobilization in young patients, severe hypercalcemia can occur [8]. Hypercalcemia following immobilization usually develops months following immobilization but can happen sooner in some cases [9]. In our case, the patient developed hypercalcemia on day 32. The mechanism is not fully understood and may be multifactorial. With the increase in osteoclastic bone resorption and the rise in sclerostin production, osteoblastic activity decreases; hence, bone formation is reduced. There is also vascular dysregulation due to denervation of the bone sympathetic neural fires. Hence, physical therapy and exercise may promote bone blood flow, alleviate bone vascular dysfunction due to neural denervation, and facilitate bone metabolism and growth [3,8,10,11].

In the case of hypercalcemia, an extensive workup is required to diagnose the cause. Workup for etiology includes obtaining serum PTH, ionized calcium, phosphorus, magnesium, alkaline phosphatase levels, and renal functions with PTHrP. Ruling out endocrine causes including adrenal insufficiency, hyperthyroidism, and pheochromocytoma is recommended. Additionally, reviewing the patient’s medication to rule out medication-induced hypercalcemia is important [6]. In the case of non-PTH-mediated hypercalcemia and after excluding malignancy and hypervitaminosis D, immobilization-induced hypercalcemia should be considered [6,12,13]. In our case, the workup was negative, and since voriconazole was suspected as a cause of hypercalcemia, we discontinued the medication. However, the calcium did not improve despite stopping the medication. Hence, we made the diagnosis of immobilization-induced hypercalcemia.

Early treatment of hypercalcemia must be focused on volume expansion and loop diuretics. Calcitonin might be used as a transient treatment initially. If no improvement, other options include denosumab initially in the case of renal dysfunction, bisphosphonates such as zoledronic acid if no improvement, or hemodialysis [5,6,12,13]. Mesland et al. described a case of immobilization-induced hypercalcemia in a patient with COVID-19 infection (calcium: 13.3 mg/dL) that was resistant to treatment and required both pamidronate and zoledronic acid to correct the hypercalcemia [5]. In our patient, hypercalcemia was resistant to hemodialysis and denosumab use. It improved after using zoledronic acid and physical therapy. Hence, we recommend early rehabilitation in all patients with COVID-19 infection. This will help achieve better functional outcomes at hospital discharge and prevent hypercalcemia induced by immobilization, especially in patients with a prolonged hospital course.

## Conclusions

COVID-19 can induce hypercalcemia caused by immobilization due to the nature of the disease and critical illness. Hypercalcemia due to immobilization in these cases can be resistant to treatment; hence, early physical therapy and rehabilitation are recommended in all patients admitted with COVID-19 to prevent such complications.
